# NKG2D modulates aggravation of liver inflammation by activating NK cells in HBV infection

**DOI:** 10.1038/s41598-017-00221-9

**Published:** 2017-03-07

**Authors:** Yadong Wang, Wei Wang, Chuan Shen, Yong Wang, Mingjing Jiao, Weiyan Yu, Hongzhu Yin, Xiaobo Shang, Qianfei Liang, Caiyan Zhao

**Affiliations:** 1 Department of Infectious Diseases, The Third Affiliated Hospital of Hebei Medical University, Shijiazhuang, China; 2 Department of Scientific Research, The Third Affiliated Hospital of Hebei Medical University, Shijiazhuang, China

## Abstract

Hepatitis B virus (HBV) infection is thought to be an immune-mediated liver disease. The mechanisms underlying natural killer (NK) cell group 2D receptor (NKG2D) that activates NK cells and participates in anti-HBV immunity and immunopathology has not been thoroughly elucidated. Peripheral NKG2D^+^ and IFN-γ^+^ NK cells frequencies and intrahepatic NKG2D and IFN-γ mRNA and protein expressions were determined in HBV-infected patients. Levels of NKG2D and IFN-γ mRNA and protein in NK cells, co-cultured with HBV-replicating HepG2 cells with or without NKG2D blockade, were analyzed. Serum and supernatant IFN-γ, TNF-α, perforin and granzyme B were measured. In results, peripheral NKG2D^+^ and IFN-γ^+^ NK cells frequencies, intrahepatic NKG2D and IFN-γ mRNA and protein levels, and serum IFN-γ, TNF-α, perforin and granzyme B levels were all highest in HBV-related acute-on-chronic liver failure group, followed by chronic hepatitis B and chronic HBV carrier groups. *In vitro*, NKG2D and IFN-γ mRNA and protein levels were higher in NK cells with IFN-α stimulation than without stimulation. Supernatant IFN-γ, TNF-α, perforin and granzyme B levels were increased under co-culture or IFN-α stimulating conditions, but were partially blocked by NKG2DmAb. In conclusion, NKG2D regulates immune inflammation and anti-viral response partly through activation of NK cells during HBV infection.

## Introduction

Host innate immunity is a key factor affecting immune recognition and viral clearance, and therefore is important for achieving sustained therapeutic effects against hepatitis B virus (HBV) infection^1^. Innate immunocytes, such as dendritic cells, natural killer (NK) and NKT cells, are important in mounting antiviral responses to HBV infection^2–6^.

Indeed, NK cells, a major component of the innate immunity, are involved in non-specific immune clearance and initiation and regulation of the specific antiviral immune response in human immunodeficiency virus (HIV) and hepatitis C virus (HCV) infection^7^. However, the frequency, distribution, and activity of NK cells in patients with chronic HBV infection has to date not been fully elucidated, and the mechanisms by which NK cells mediate antiviral response remain unclear. Preliminary data indicate that the frequency and distribution of NK cells in peripheral blood and hepatic tissue are amplified during the immune clearance phase of chronic hepatitis B (CHB), especially the frequency of natural killer cell group 2D receptor positive (NKG2D^+^) NK cells in the liver^8^. However, Peppa *et al*.^9^ demonstrated that NK cells only negatively regulate antiviral immunity in chronic HBV infection and illustrated a novel mechanism of T cell tolerance in the human liver. Other studies also suggested that HBV replication may have differential effects on NK cell ligands, potentiating escape mechanisms through down-regulation of major histocompatibility complex class I polypeptide-related chain A (MICA), which is a NKG2D ligand. Upregulation of NK cell ligands can be countered by decreasing MICA, and weakening NK surveillance^10^.

NKG2D receptor, a C lectin like receptor of NKG2 family, is expressed primarily by NK cells, γδ T cells and CD8^+^ αβ T cells^11, 12^ and is a potent non-classical MHC molecule regulating receptor on NK cell membrane, which plays an important role in supplementing the function of natural cytotoxicity receptor (NCR). Although the integrative mechanism of NK cells in antitumor and antiviral responses are not fully understood, NKG2D appears to be play a key role and is, therefore, an attractive therapeutic target^13^. During HBV infection, increasing evidence shows that NKG2D-ligand interactions are critical in the establishment of HBV persistent infection, the development of liver injury and HCC^14^. It has been reported that HBsAg transgenic mice (HBs-B6) are more sensitive to Poly I:C induced liver damage due to hepatic infiltration of NK cells and the increased production of interferon gamma (IFN-γ)^15^. Another study based on HBV transgenic mice (HBs-Tg) indicated that severe liver damage induced by low dose CoA is also dependent on intrahepatic NK cells infiltration and activation mediated by NKG2D^16^. Similarly, the liver failure mouse model established by murine hepatitis virus strain 3 (MHV-3) also showed that NK cell mediated hepatocyte damage could be partially alleviated by blocking NKG2D or its ligand signaling pathway^17, 18^. These studies suggested a possible interaction between HBV and NKG2D, in which HBx and HBc proteins may down-regulate the NKG2D receptor activity of NK cells and reduce cytotoxicity and IFN-γ secretion, leading to a reduction in NK cell mediated antiviral immunoreactivity^19^. However, it is not clear how NKG2D activated NK cells function to deliver viral clearance and contribute to hepatic injury in HBV infected patients. In the current study, frequency of NKG2D^+^ NK cells in peripheral blood mononuclear cells (PBMCs), intrahepatic expression of NKG2D mRNA and protein, as well as IFN-γ, TNF-α, perforin and granzyme B were investigated in HBV infected patients at different immunological stages. Furthermore, the cytotoxicity of NK cells on HBV replicating HepG2 cells was analyzed. The regulatory effect of NKG2D mediated NK cells activation on antiviral response was also assessed.

## Results

### Demographic and Clinical Characteristics

A total of 45 chronic HBV-infected patients and 15 blood/liver donors (37 males and 23 females) were enrolled in this study. Mean age was 38 ± 12 years (range: 16–65 years). All subjects were comparable in both age and sex. The HBV-ACLF patients (IH, immune hyperactivation) displayed significantly higher levels of ALT, TBil, but lower PTA levels than CHB patients (IA, immune activation), chronic HBV carriers (IT, immune tolerance), and healthy controls (HCs, all *P* < 0.01). Furthermore, the HBV-ACLF patients also displayed relatively lower levels of serum HBV DNA and HBsAg than CHB patients (*P* < 0.05) (Table 1). All ACLF patients were at end stage of liver disease. Among them, one had hepatorenal syndrome, four had ascites, and one had hepatic encephalopathy. However, no infection and GI bleeding existed in any patients.

**Table 1:** Demographic and clinical characteristics of all recruited subjects in this study (Mean ± SD).

Characteristic	HCs	Chronic HBV carriers (IT)	CHB (IA)	HBV-ACLF (IH)
n	15	15	15	15
Mean age, years	38 ± 12	36 ± 6	35 ± 10	45 ± 16
Male/Female, n	8/7	9/6	10/5	10/5
Serum ALT, U/L	22 ± 6	28 ± 6	180 ± 49^a,c^	251 ± 52^a,c,e^
Serum TBil, μmol/L	9.6 ± 4.5	11.3 ± 7.5	75.2 ± 39.5^b,d^	296.5 ± 145.3^a,c,e^
Albumin, g/L	41.6 ± 4.5	39.5 ± 4.8	36.4 ± 6.3^b^	32.2 ± 5.4^a,c,f^
PTA, %	—	100.8 ± 11.2	96.6 ± 20.1	28.5 ± 9.2^b,d^
HBV DNA, Log_10_ copies/mL	—	7.17 ± 0.87	5.07 ± 0.54^b^	4.72 ± 0.71^c^
Serum HBeAg, S/Co	—	356.25 ± 169.05	251.88 ± 228.43^c^	224.58 ± 134.55^c,e^
Serum HBsAg, IU/mL	—	6135.59 ± 3542	5450.86 ± 1958	3468.45 ± 2698^d^

ALT, alanine aminotransferase; TBil, serum total bilirubin; PTA, prothrombinase activity; HBV hepatitis B virus; HCs, healthy controls; chronic HBV carriers (IT); CHB, chronic hepatitis B (IA); HBV-ACLF, HBV-related acute-on-chronic liver failure (IH).

Compared with HCs group, ^a^ *P* < 0.01, ^b^ *P* < 0.05; Compared with ITgroup, ^c^ *P* < 0.01, ^d^ *P* < 0.05; Compared with IAgroup, ^e^ *P* < 0.01, ^f^ *P* < 0.05.

### Percentages of peripheral NK, NKG2D^+^ NK, and IFN-γ^+^ NK cells

Compared with the healthy controls (13.58 ± 3.24%), the frequency of peripheral NK cells was lower in each chronic HBV-infected group, while the frequency was higher in both CHB (8.43 ± 2.92%) and HBV-ACLF groups (7.92 ± 2.85%) than chronic HBV carriers (5.42 ± 2.18%, all *P* < 0.05). No difference was detected between HBV-ACLF and CHB group (*P* > 0.05).

**Figure 1:**
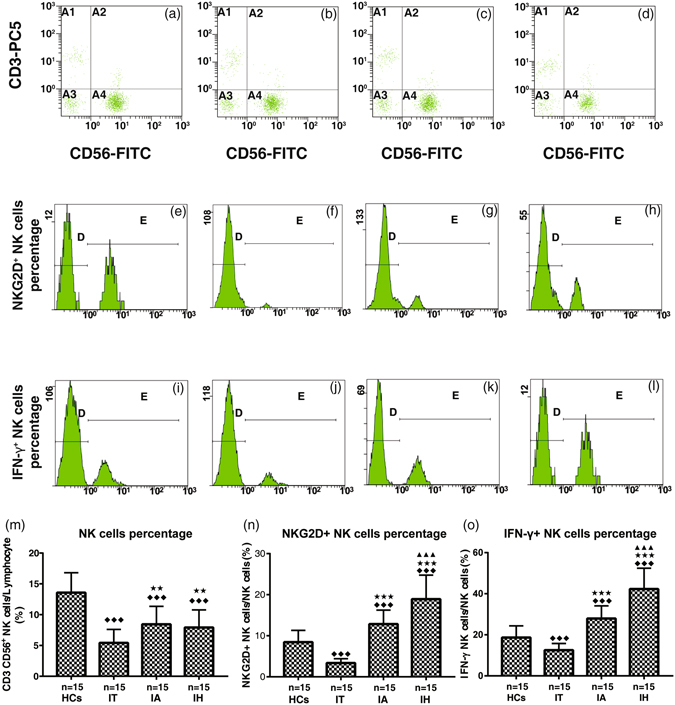
Percentages of NK (CD3^−^ CD56^+^) cells (**a**–**d**, **m**) in PBMC, NK cell group 2D receptor (NKG2D)^+^ (**e**–**h**, **n**) and IFN-γ^+^ (**i**–**l**, **o**) NK cells within total NK cells. HCs, healthy controls; IT, chronic HBV carriers; IA, CHB patients; IH, HBV-ACLF patients. Student-Newman-Keuls *q* test following one-way ANOVA were used for comparing percentages of NK cells, and Nemenyi test following Kruskal-Wallis *H* test were used for comparing percentages of NKG2D^+^ and IFN-γ^+^ NK cells between two groups. Compared with HCs group, ^◆◆◆^ *P* < 0.01; Compared with IT group, ^★★★^ *P* < 0.01, ^★★^ *P* < 0.05; Compared with IA group, ^▲▲▲^ *P* < 0.01.

On the contrary, the percentage of NKG2D^+^ and IFN-γ^+^ NK cells in HBV-ACLF group were the highest (18.92 ± 5.85% and 42.25 ± 10.17%) followed by CHB group (12.85 ± 3.39% and 27.95 ± 6.12%), healthy controls (8.45 ± 2.86% and 18.69 ± 5.68%) and chronic HBV carriers (3.36 ± 1.05% and 12.55 ± 3.24%), and the difference was significant between any two groups (*P* < 0.01 or <0.05) (Fig. 1a and b).

### Intrahepatic NKG2D and IFN-γ mRNA expression

**Figure 2:**
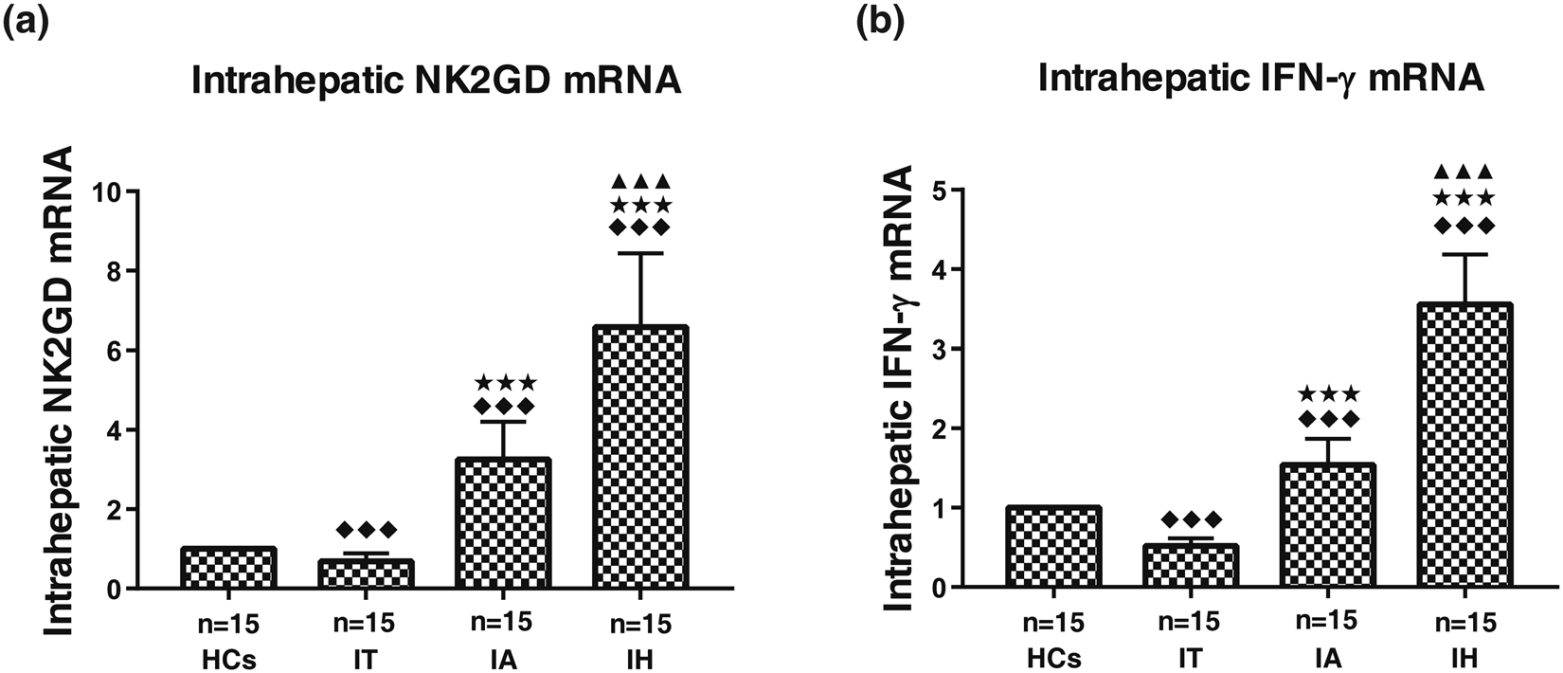
Intrahepatic expression of NKG2D mRNA (**a**) and IFN-γ mRNA (**b**). HCs, healthy controls (the relative expression were defined as 1.00); IT, chronic HBV carriers; IA, CHB patients; IH, HBV-ACLF. Nemenyi test following Kruskal-Wallis *H* test were used for comparing mRNA expressions of NKG2D and IFN-γ between two groups. Compared with HCs group, ^◆◆◆^ *P* < 0.01; Compared with IT group, ^★★★^ *P* < 0.01; Compared with IA group, ^▲▲▲^ *P* < 0.01.

Intrahepatic mRNA levels of NKG2D and IFN-γ were analyzed by real-time quantitative RT-PCR in liver biopsy samples. The relative mRNA levels of NKG2D (6.58 ± 1.86) and IFN-γ (3.56 ± 0.63) were the highest in HBV-ACLF patients, followed by CHB (3.25 ± 0.95; 1.54 ± 0.33) and chronic HBV carriers (0.69 ± 0.20; 0.52 ± 0.09) patients. The difference between any two groups was statistically significant (all *P* < 0.01) (Fig. 2a and b).

### Intrahepatic NKG2D and IFN-γ protein expression

**Figure 3:**
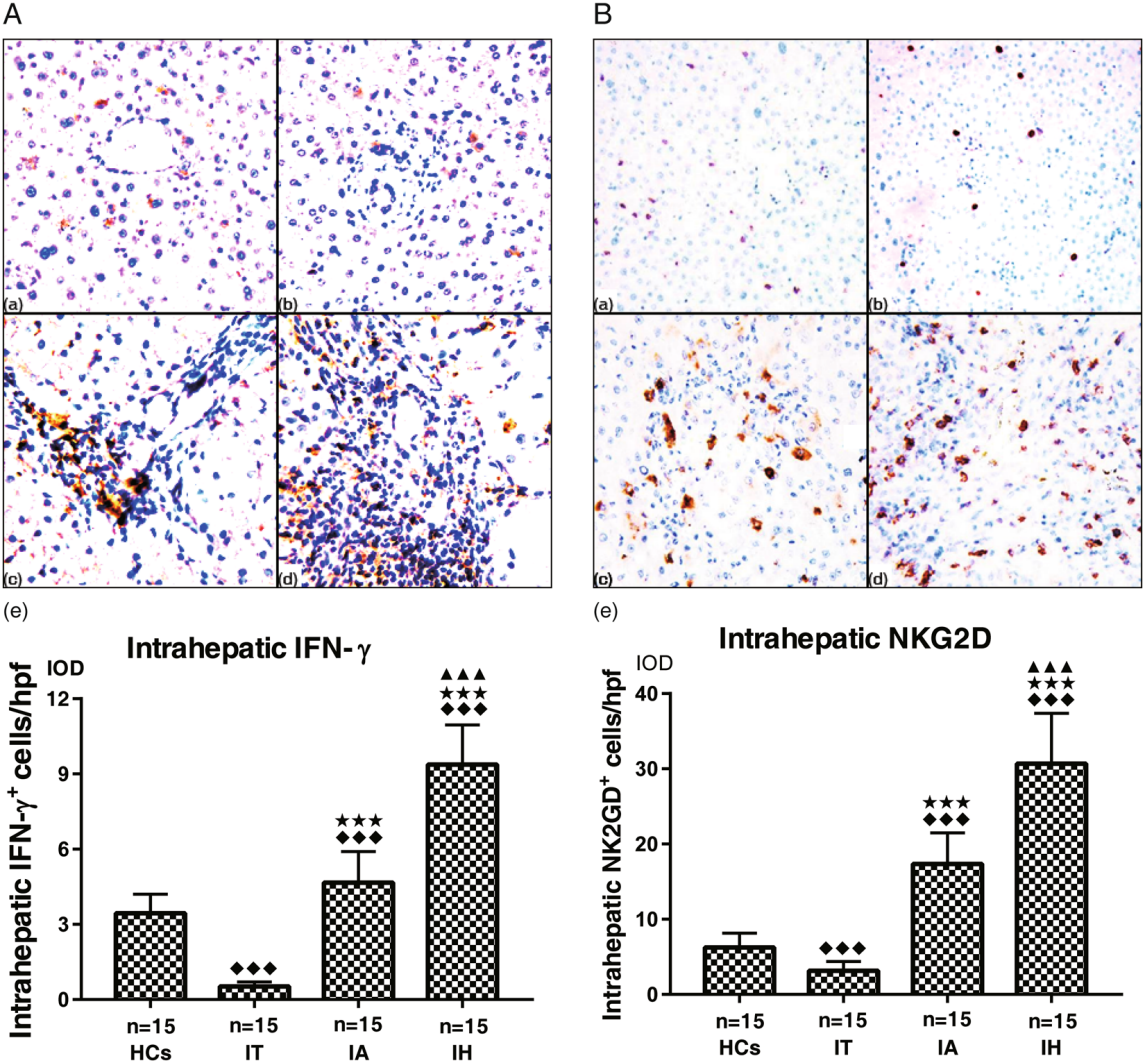
Representative graphs of intrahepatic IFN-γ^+^ cells (A, 200×) and NKG2D^+^ cells (B, 200×) expressions. (**a**) HCs, healthy controls, (**b**) IT, chronic HBV carriers, (**c**) IA, CHB patients, (**d**) IH, HBV-ACLF patients. (**e**) Collective analysis of results from all 4 groups. IFN-γ^+^ cells were distributed mainly in the inflammatory sites and periportal areas that were infiltrated with lymphocytes. NKG2D^+^ cells were mainly distributed in Disse’s space of hepatic lobule in HCs and chronic HBV carriers, and mainly in periportal areas in CHB and HBV-ACLF group. Nemenyi test following Kruskal-Wallis *H* test were used for comparing intrahepatic IFN-γ^+^ and NKG2D^+^ cells expressions between two groups. Compared with HCs group, ^◆◆◆^ *P* < 0.01; Compared with IT group, ^★★★^ *P* < 0.01; Compared with IA group, ^▲▲▲^ *P* < 0.01.

We performed immunohistochemistry (IHC) staining to semi-quantify hepatic infiltration of NKG2D^+^ and IFN-γ^+^ cells. As shown in Fig. 3, IFN-γ^+^ cells were distributed mainly in the inflammatory sites and periportal areas that were infiltrated with lymphocytes. NKG2D^+^ cells were mainly distributed in Disse’s space of hepatic lobules in healthy controls and the chronic HBV carriers, but mainly in periportal areas in CHB and HBV-ACLF groups. Semi-quantitative analysis revealed the strongest expression of NKG2D^+^ and IFN-γ^+^ was detected in the HBV-ACLF patients (30.69 ± 6.67; 9.52 ± 2.12), followed by CHB patients (17.36 ± 4.13; 5.54 ± 1.62) and HCs (6.24 ± 1.93; 3.26 ± 0.85), and the weakest in chronic HBV carriers (3.16 ± 1.24; 1.02 ± 0.59). The differences between each two groups were statistically significant (all *P* < 0.05).

### NKG2D and IFN-γ mRNA and protein expressions in NK+ HBV-HepG2 co-cultured system

**Figure 4:**
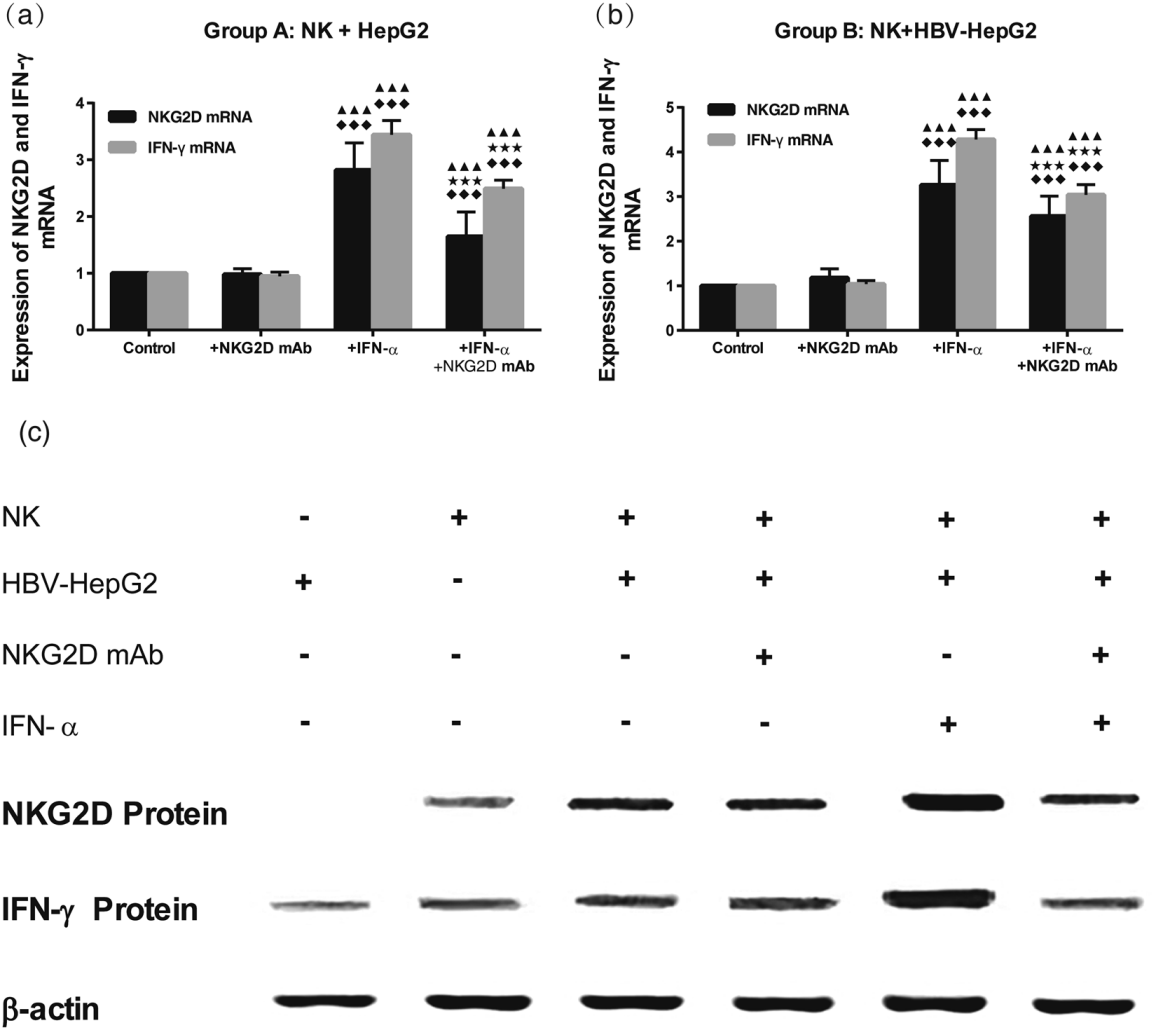
Analysis of NKG2D and IFN-γ mRNA levels in co-cultured cells (NK + HepG2/HBV-HepG2) of Group A (**a**, NK + HepG2) and Group B (**b**, NK + HBV-HepG2). Nemenyi test following Kruskal-Wallis *H* test were used for comparing mRNA expressions of NKG2D and IFN-γ between two compared groups. Compared with Control group (NK + HepG2 or NK + HBV-HepG2), ^◆◆◆^ *P* < 0.01; Compared with Control + NKG2D mAb group, ^★★★^ *P* < 0.01; Compared with Control + IFN-α group, ^▲▲▲^ *P* < 0.01; Analysis of the levels of NKG2D and IFN-γ protein in different groups (**c**). The density of NKG2D and IFN-γ protein was the highest in group of NK + HBV-HepG2 + IFN-α, followed by group of NK + HBV-HepG2 + IFN-α + NKG2DmAb and group of NK + HBV-HepG2 + NKG2DmAb.

In group B of NK+ HBV-HepG2, the relative levels of NKG2D and IFN-γ mRNA in sub-group of +IFN-α (3.26 ± 0.55; 4.28 ± 0.22) were the highest as detected by qPCR, followed by sub-group of +IFN-α + NKG2DmAb (2.56 ± 0.45; 3.04 ± 0.23) and sub-group of +NKG2DmAb alone (1.18 ± 0.20; 1.04 ± 0.08). The differences between each two groups were statistically significant (all *P* < 0.05). In group A of NK + HepG2, the same change trends and statistical differences were detected (all *P* < 0.05) (Fig. 4a and b).

Relative NKG2D and IFN-γ protein levels in co-cultured cells were detected by Western Blot. The density of both NKG2D and IFN-γ proteins in the sub-group of +IFN-α was the highest, followed by sub-group of +IFN-α +NKG2DmAb and sub-group of +NKG2DmAb alone. The differences between each two groups were statistically significant (all *P* < 0.05) (Fig. 4c).

### HBV DNA level in cell supernatant of NK+ HBV-HepG2 co-cultured system

HepG2cells transfected with wide type HBV pCH-9/3093 plasmid DNA support a stable HBV replication (6.9 × 10^6^ copies/mL) and HBsAg expression (72.62 ± 10.35 IU/mL). HBV DNA and HBsAg levels were decreased slightly in the single cultured system of HBV-HepG2 cells under IFN-α treatment (9.2 × 10^5^ copies/mL; 69.45 ± 6.96 IU/mL), as well in co-cultured system of NK+ HBV-HepG2 cells without IFN-α treatment (5.5 × 10^5^ copies/mL: 52.43 ± 5.96 IU/mL; all *P* > 0.05). However, IFN-α significantly inhibited HBV DNA replication and HBsAg expression in the co-culture system (3.2 × 10^3^ copies/mL; 36.45 ± 6.28 IU/mL) as compared to no IFN-α treatment. This was partly blocked by NKG2DmAb (4.6 × 10^5^ copies/mL; 42.62 ± 8.95 IU/mL). The differences between the two groups were statistically significant (all *P* < 0.05) (Table 2).

**Table 2:** Levels of IFN-γ, TNF-α, perforin and granzyme B in different co-cultured supernatants after addition of IFN-α alone or together with NKG2D mAb (Mean ± SD).

Group	n	TNF-α (pg/mL)	IFN-γ (pg/mL)	perforin (pg/mL)	granzyme B (pg/mL)	HBV DNA (copies/mL)	HBsAg (IU/mL)
HepG2 cells							
Control	6	6.83 ± 0.58	0.12 ± 0.02	—	—	—	—
+IFN-α	6	14.95 ± 2.85^a^	0.15 ± 0.05	—	—	—	—
HBV- HepG2 cells							
Control		8.63 ± 1.45	0.14 ± 0.03	—	—	6.9 × 10^6^	72.62 ± 10.35
+IFN-α		18.85 ± 2.88^a^	0.18 ± 0.06	—	—	9.2 × 10^5^	69.45 ± 6.96
NK cells							
Control	6	12.45 ± 1.25	0.34 ± 0.07	45.89 ± 8.65	35.25 ± 3.19	—	—
+IFN-α	6	86.58 ± 8.69^a^	1.28 ± 0.12^a^	82.69 ± 13.69^a^	52.43 ± 5.46^a^	—	—
+NKG2D mAb	6	10.36 ± 1.33	0.32 ± 0.02	36.79 ± 11.65	32.28 ± 2.93	—	—
+IFN-α + NKG2D mAb	6	62.65 ± 6.58^a,b,c^	0.87 ± 0.08^a,b,c^	58.95 ± 9.69^b,c^	40.88 ± 5.23^a,b,c^	—	—
Group A: NK + HepG2							
Control	6	45.65 ± 3.58	67.25 ± 6.45	56.89 ± 8.25	42.56 ± 4.93	—	—
+IFN-α	6	98.95 ± 9.46^a^	154.32 ± 15.30^a^	96.25 ± 12.33^a^	72.55 ± 9.42^a^	—	—
+NKG2D mAb	6	39.86 ± 3.25	58.49 ± 5.68	32.89 ± 7.98^a^	36.25 ± 4.14	—	—
+IFN-α + NKG2D mAb	6	69.50 ± 5.92^a,b,c^	122.06 ± 13.25^a,b,c^	75.58 ± 7.68^a,b,c^	58.44 ± 5.88^a,b,c^	—	—
Group B: NK + HBV-HepG2							
Control	6	65.28 ± 4.68	82.39 ± 9.88	65.85 ± 10.89	58.26 ± 6.13	5.5 × 10^5^	52.43 ± 5.96
+IFN-α	6	125.45 ± 1.85^a^	215.24 ± 18.13^a^	118.25 ± 15.85^a^	82.50 ± 9.14^a^	3.2 × 10^3^	36.45 ± 6.28
+NKG2D mAb	6	48.85 ± 4.58^a^	57.86 ± 7.25^a^	42.55 ± 8.62^a^	41.25 ± 4.45^a^	7.8 × 10^6^	72.18 ± 8.69
+IFN-α + NKG2D mAb	6	88.45 ± 8.59^a,b,c^	138.26 ± 11.62^a,b,c^	75.86 ± 11.25^a,b,c^	66.58 ± 7.24^a,b,c^	4.6 × 10^5^	42.62 ± 8.95

NKG2D, NKG2 family receptor D; IFN, interferon; TNF, tumor necrosis factor; HBV hepatitis B virus; HBsAg: surface antigen of hepatitis B; ^a^ *P* < 0.05, compared with the control group of the same co-cultured system. ^b^ *P* < 0.05, compared with NK + IFN-α group of the same co-cultured system. ^c^ *P* < 0.05, compared with NK + NKG2D mAb group of the same co-cultured system.

### IFN-γ, TNF-α, perforin and granzyme B levels in serum and cell supernatant

**Figure 5:**
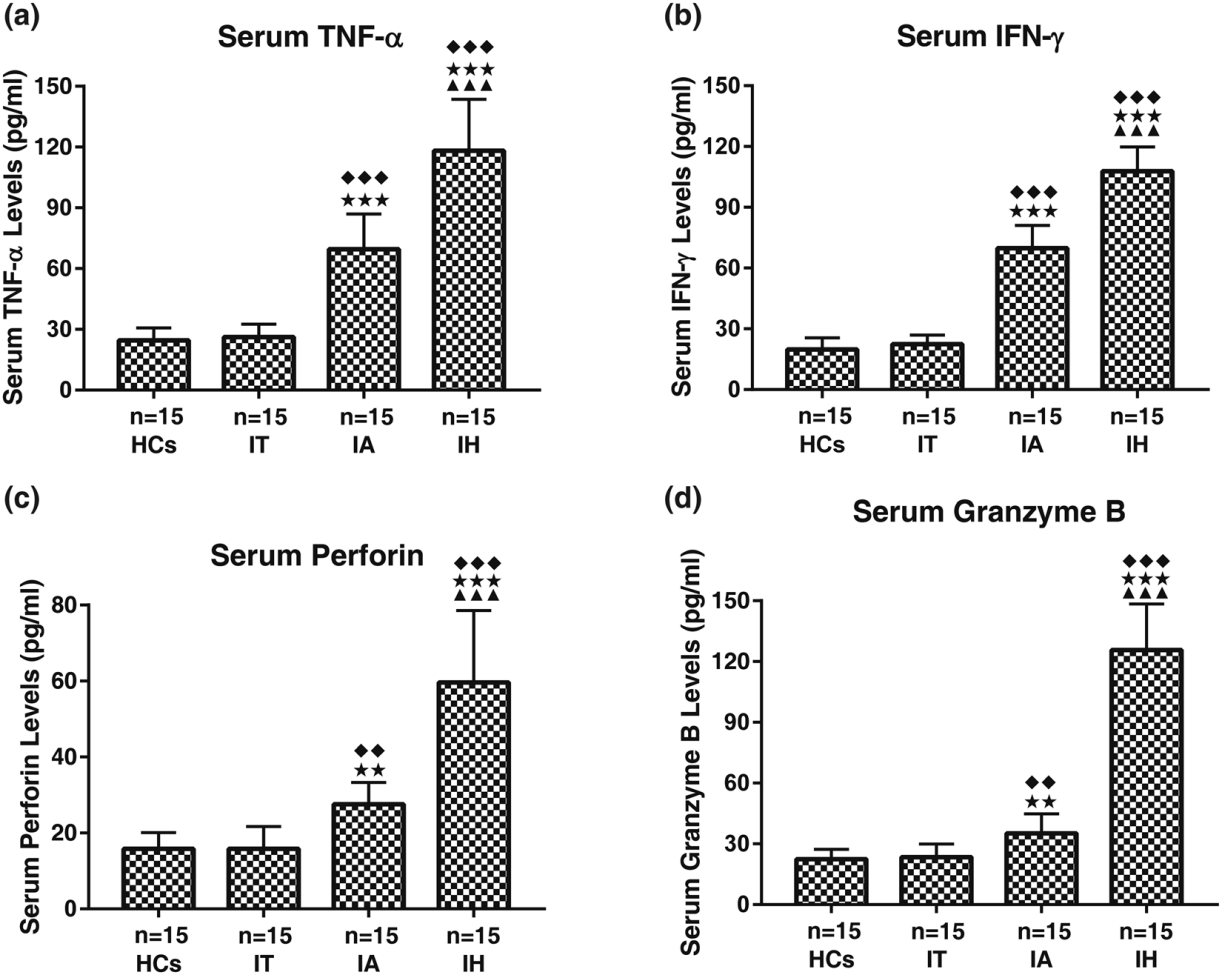
Serum Levels of IFN-γ, TNF-α, perforin and granzyme B in different clinical stages of chronic HBV-infected patients. HCs, healthy controls; IT, chronic HBV carriers; IA, CHB patients; IH, HBV-ACLF. Nemenyi test following Kruskal-Wallis *H* test were used for comparing IFN-γ, TNF-α, perforin and granzyme B levels between two compared groups. Compared with HCs group, ^◆◆◆^ *P* < 0.01, ^◆◆^ *P* < 0.05; Compared with IT group, ^★★★^ *P* < 0.01, ^★★^ *P* < 0.05; Compared with IA group, ^▲▲▲^ *P* < 0.01.

We examined serum levels of IFN-γ, TNF-α, perforin and granzyme B in all enrolled subjects by ELISA. Patients with HBV-ACLF had a significantly higher levels of serum IFN-γ, TNF-α, perforin and granzyme B than CHB, chronic HBV carriers and healthy controls (all *P* < 0.01 or <0.05). There were significantly statistical differences between each two groups except chronic HBV carriers and healthy controls (all *P* < 0.01 or <0.05) (Fig. 5).

**Figure 6:**
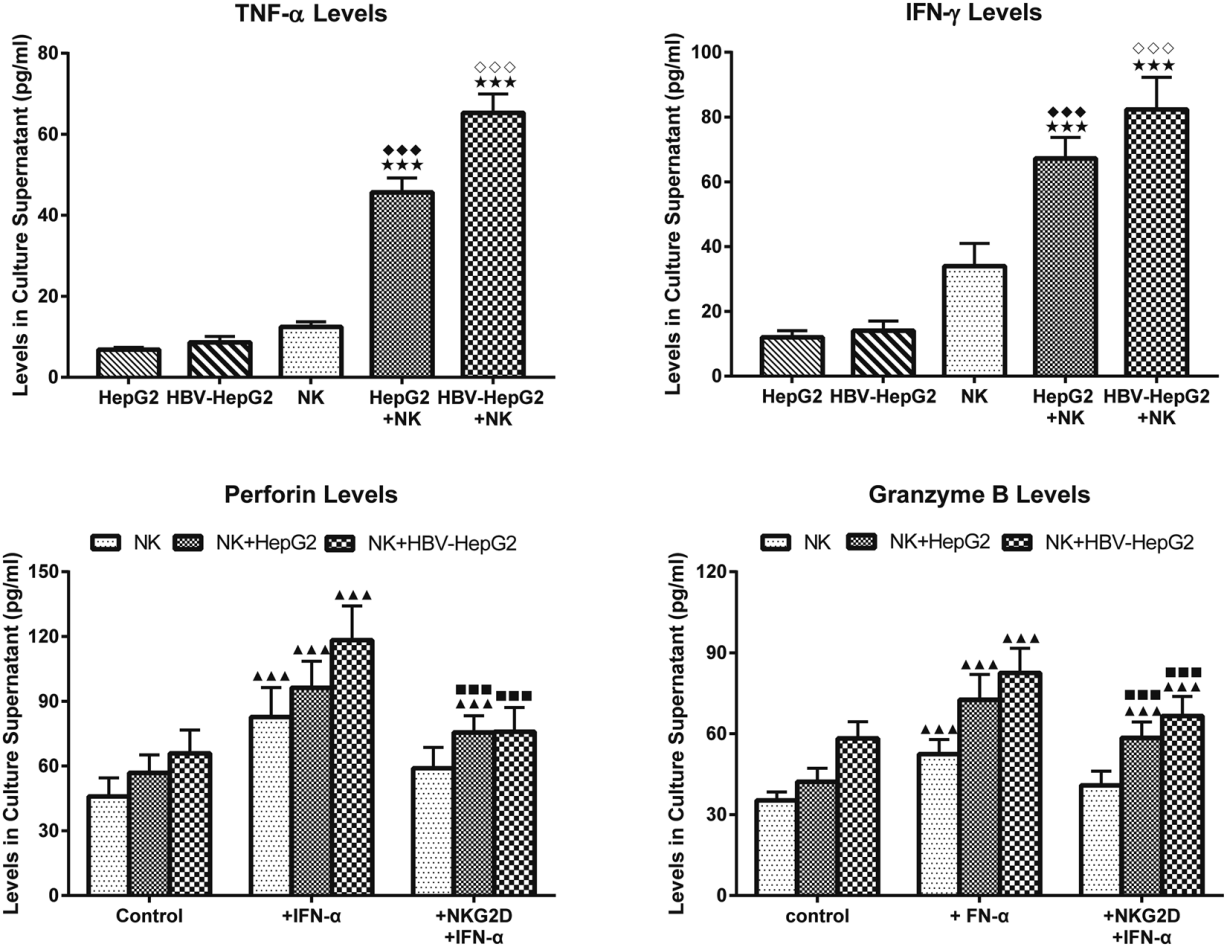
Levels of TNF-α (**a**) and IFN-γ (**b**) in the supernatants with or without co-cultured NK cells. Student-Newman-Keuls *q* test following one-way ANOVA were used for comparing IFN-γ, TNF-α, perforin and granzyme B levels between two compared groups. Compare with HepG2 cells group, ^◆◆◆^ *P* < 0.01; Compare with HBV-HepG2 cells group, ^◇◇◇^ *P* < 0.01; Compare with NK cells group, ^★★★^ *P* < 0.01. Levels of perforin (**c**) and granzyme B (**d**) in the supernatants in the presence IFN-α with or without NKG2D blocking. Compare with control group, ^▲▲▲^ *P* < 0.01; Compare with + IFN-α group, ^■■■^ *P* < 0.01.

The expression of TNF-α, IFN-γ, perforin and granzyme B in the co-culture group was higher than the single-cultured group (all *P* < 0.01). IFN-α stimulation significantly elevated the expression of these cytokines (all *P* < 0.01), especially in the co-culture system. However, inclusion of anti NKG2DmAb significantly inhibited the activation of NK cells induced by IFN-α (all *P* < 0.01), although the blocking efficacy cannot restore these indexes to the levels of pre-intervention (Table 2, Fig. 6).

## Discussion

In this study, we found that the frequency of NK cells in PBMC and hepatic tissues was increased in CHB patients who were at the immune activation stage, and in the HBV-ACLF patients with excessive immune activation, as compared with chronic HBV carriers at the stage of immune tolerance. Furthermore, intrahepatic infiltration of NK cells was significantly higher, but the frequency of NK cells in PBMC was lower in HBV-ACLF patients compared with CHB patients, which suggested that NK cells were recruited to the liver for participation in immune regulation and hepatic immune injury, which were suggested in HCV infection^20^ and liver failure in mice model^17^. To verify antiviral effects, we established the NK and HBV-HepG2 cells co-culture systems. However, because of the limited volumes of blood samples and the difficulty of *in vitro* amplification of detached primary NK cells, we were only able to use the cell line NK-92 as a succedaneum in this study^21, 22^. Activation of NK cells in chronic HBV infection is a “double-edged sword”: moderate activation is thought to be beneficial to breaking immune tolerance and balancing antiviral intensity, but excessive immune activation may cause pathological damage and thus increase the risk of liver failure^23, 24^. Unfortunately, in light of multiple factors involved in HBV infection pathogenesis, a satisfactory cut-off range for the number of activated NK cells that can distinguish a beneficial from a harmful effect has not yet been established.

The strength of immune response exerted by NK cells is dependent on both the number and status of NK cells. Multiple functional receptors, including NCR, NKG2 family receptors, NKp30, NKp46, are expressed on the surface of NK cells though different NK cell subtypes differ in receptors type and expression levels. Activation and density of these receptors determines the antiviral cytotoxicity of NK cells. Recently, the role of NKG2 family receptors, especially NKG2D, in HBV infection pathogenesis is a focus of research by hepatologists^16, 17, 25, 26^. In the current study, our findings that the frequency of NKG2D^+^ NK cells in PBMC, and the intrahepatic expression of NKG2D mRNA and protein were significantly increased in patients with CHB, especially HBV-ACLF. These results are consistent with the previously published results^27^, which indicate that the over-expression and activation of NKG2D may facilitate NK cell mediated cytotoxicity and immune injury to HBV infected liver. However, there is no general consensus regarding studies in the role of NK, NKG2D and HBV^9^. A recent study suggested that patients with HBV-ACLF demonstrated fewer peripheral NK cells, although this was not significant compared to other groups. Activated NKG2D receptors were increased in patients with HBV-ACLF, however, the function of NK cells, including cytotoxicity and production of INF-γ and TNF-α, were both downregulated in patients with HBV-ACLF and CHB due to increased inhibitory receptors, such as CD158a^28^. Killing of HBV infected hepatocytes by NK cells, which may involve perforin/granzyme B mediated cytotoxicity, also secrete IFN-γ and TNF-α, as well as stimulating hepatocytes, Kupffer cells and sinusoid endothelial cells to secrete CXC chemokine ligand, recruiting other immunocytes to infiltrate into the liver.

It has been indicated that NK cells participate in the pathological process of acute liver failure in mice infected with MHV-3, and the blockade of NKG2D receptor could reduce hepatocyte injury to a certain degree^17^. Using siRNA in HBs-Tg mice also showed that NKG2D activated NK cells were associated with fulminant hepatic injury induced by ConA, but mice treated with RNAi against NKG2D ligand were protected from ConA induced liver injury^29^. An *in vitro* study by Liu *et al*. showed both HBx and HBc proteins could also interfere with cytotoxicity and IFN-γ secretion of NK cells by down-regulating NKG2D on NK cell surface^19^. In the current study, the co-culture of NK cells with HBV-HepG2 cells showed that the activity of NK cells was greatly enhanced, following the secretion of TNF-α, IFN-γ, perforin and granzyme B. Furthermore, NKG2D mRNA/protein levels were increased by nearly two fold after IFN-α treatment in co-culture, which was associated with the inhibition of HBV DNA replication in HBV-HepG2 cells. However, blockade of the NKG2D signal pathway by NKG2DmAb resulted in a decrease in the cytotoxicity of NK cells induced by IFN-α. Therefore, effective regulation of NKG2D could modulate NK cell mediated immune response and liver injury. Blockade of NKG2D with an antibody cannot fully block NK cell cytotoxicity, but can restore the serum cytokines to baseline levels, indicating that there might be other pathways involved, such as NKG2A, NKG2C, NKp30, or NKp46, regulating NK cell cytotoxicity and NKG2D activation^7, 30^.

Our study verified the role of NKG2D activity is of important significance in regulating liver injury and controlling anti-HBV replication mediated by NK cells, although inevitably carries some limitations. Firstly, although NKG2D is mainly expressed by NK cells, it is also expressed by NKT cells, γδ T cells and CD8^+^ αβ T cells. Because the percentage of NK cells in the liver is approximately 35%, which is further expanded upon HBV infection, we believe that our study used a representative sample of lymphocytes from the liver. Secondly, due to limitations in harvesting liver tissues, this study was unable to analyze cell subtypes in a highest resolution. Furthermore, we did not distinguish CD56dim from CD56bright NK cells because of the relatively lower frequency of NKG2D^+^ NK cells subtype. Indeed, there was no difference in the expression of NKG2D in cultured PBMC between two subtypes identified by Takahashi *et al*.^31^. Thirdly, we only detected mRNA levels, which may not always proportionally predict the protein levels. In conclusion, the NKG2D receptor plays an important role in influencing the outcomes of HBV infection even though the exact mechanisms involved remain be established. Our study confirmed that blocking NKG2D with an antibody can alleviate the cytotoxic effect of NK cell and reduce inhibition of HBV replication. Further investigations will focus on the delineation of the dynamic alteration of NK cells, NKG2D^+^, and IFN-γ^+^ NK cells during the processes treating CHB and HBV-ACLF patients, association between each measurement and clinical outcome of the treatment, and the detailed signaling pathways and regulated targets of NKG2D activation, with the ultimate objective of elucidating the principle of NKG2D activated NK cell and its potential value for antiviral therapy.

## Materials and Methods

### Clinical Samples

In total, 60 subjects were enrolled in this prospective cohort study. Three groups of 15 chronic hepatitis B (CHB) patients (IA, immune activation), 15 chronic HBV carriers (IT, immune tolerance), and 15 patients with HBV-related acute-on-chronic liver failure (HBV-ACLF) (IH, immune hyperactivation) were recruited from January 2010 to December 2012 in the Third Affiliated Hospital of Hebei Medical University. All patients were diagnosed in accordance with the guidelines for the management of CHB (2008 update) and the consensus recommendation for ACLF criteria (2009) by the Asian Pacific Association for the Study of the Liver (APASL)^32, 33^. All the included patients met the following criteria: (1) Chronic HBV carrier (IT group): HBsAg- and HBV DNA- positive, either HBeAg-positive, and persistently normal ALT and AST levels for at least 3 times over one year period or the documented normal liver histology. (2) CHB patients (IA group): HBsAg- and HBV DNA-positive, either HBeAg-positive or anti-HBe-positive, persistently or intermittently elevated ALT levels or histologic evidence of liver injury. (3) HBV-ACLF patients (IH group): diagnosed by both clinical and histopathologic criteria, in addition to HBsAg-and HBV DNA-positive, either HBeAg or anti-HBe-positive. All patients did not receive antiviral or immunotherapy treatment six months prior to sampling. Serum samples from 15 healthy donors were collected as healthy controls. Liver tissue specimens from chronic HBV carriers and CHB patients were obtained by routine echo-guided percutaneous needle biopsy, while liver tissues from HCs and HBV-ACLF patients were obtained from donors and recipients during liver transplantation. Exclusion criteria for patients included co-infections with HAV, HCV, HEV, Epstein-Barr virus, cytomegalovirus, HIV; drug-induced liver injury; alcoholic or non-alcoholic fatty liver disease; autoimmune liver disease; hepatocellular carcinoma and pregnant women.

The study protocol was approved by the Human Research Ethics Committee of Hebei Medical University, China. All procedures performed in studies involving human participants were in accordance with the ethical standards of the institutional and national research committee and with the 1964 Helsinki declaration and its later amendments or comparable ethical standards. All the patients provided written informed consent to participate in the study, which included a review of each subject’s medical records.

### Flow Cytometry Analysis

PBMCs were isolated from freshly heparinized blood by density gradient centrifugation using Ficoll-Hypaque. The cells were stained with FITC conjugated anti-human CD56, PE-Cy5 conjugated anti-human CD3, and APC conjugated anti-human NKG2D antibody (BioLegend, San Diego, CA, USA), and then were analyzed by flow cytometry (EPICS XL-4, Beckman-Coulter Inc., Brea, CA, USA). NK (CD3^−^CD56^+^) cells were gated within the lymphocyte population while NKG2D^+^ cells were gated within the NK cells.

In addition, fresh PBMCs were incubated with RPMI 1640 medium (Invitrogen, Carlsbad, CA, USA) supplemented with 10% fetal calf serum (Hyclone South Logan, UT, USA) at 37 °C, 5% CO_2_ for 4 h in the presence of 50 ng/mL phorbol 12-myristate 13-acetate (PMA), 1 μg/mL ionomycin, and 1.5 μg/mL Monensin (Sigma, St. Louis, MO, USA). Cells were stained with PE-Cy5 conjugated anti-human CD3 and FITC conjugated anti-human CD56 antibody, and then fixed and permeabilized using IntraPrep TM Kit (Beckman-Coulter, Brea, CA, USA). Next, the cells were stained with PE conjugated anti-human IFN-γ antibody (BioLegend, San Diego, CA, USA), and then analyzed by flow cytometry. IFN-γ^+^ cells were gated within the NK cells.

### Immunohistochemistry Staining

Liver tissues samples were fixed in 4% buffered formaldehyde, embedded into paraffin blocks, and sliced (5 μm) for subsequent liver pathology with hematoxylin and eosin (H&E) and immunohistochemistry assays. The immunohistochemistry (IHC) staining was performed following a standard protocol of Power VisionTM Two-Step Detection System in our laboratory, as described previously^34^. Briefly, antigen retrieval was achieved via pressure cooking for 15 min in citrate buffer (pH 6.0) and blockade of endogenous peroxidase activity was achieved with 3% H_2_O_2_ for 15 min. Slides were incubated with polyclonal rabbit-anti-human NKG2D antibody (1:200 dilution, Abcam, Cambridge, MA, USA) and IFN-γ antibody(1:200 dilution, Boster Biotechnology, Wuhan, China) overnight at 4 °C. Tissues were then incubated with goat-anti-rabbit secondary antibody (ZSGB Biotech, Beijing, China) for 40 min at 37 °C after washing with phosphate-buffered saline and sections were visualized using a 3,30-diaminobenzidine substrate kit (ZSGB Biotech, Beijing, China) followed by counterstaining with haematoxylin. Brown-yellow cellular staining indicated positive expression. The immunoreactivity was quantified by outlining the whole liver sections using Image Pro Plus software (Media Cybernetics, Silver Spring, MD, USA) to determine the values of integrated optical density (IOD).

### Plasmid Transfection and Efficiencies Evaluation

Newly revived HepG2 cells (provided by Professor Dianxing Sun, Bethune International Peace Hospital of Chinese PLA) were plated and cultured at 5% CO_2_, 37 °C in 6-well culture plates (5 × 10^5^ cells/well) with high glucose-DMEM medium (Gibco, Grand Island, NY, USA) containing 10% FBS, penicillin (100 U/mL), and streptomycin (100 μg/mL). For transfection, cells were seeded into six-well tissue culture plate 24 h prior to transfection. When the cells reached the logarithmic phase with 60–70% confluence, fresh complete medium was replaced and HepG2 cells were transfected with 8 μg of plasmid DNA (wide type HBV pCH-9/3093, a gift of Professor Dianxing Sun)^35, 36^ using VigoFect transfection kit (Vigorous Biotechnology, Beijing, China) per manufacturer’s recommended protocol^37^. Transfection medium was replaced with complete HD-DMEM after 3–12 h incubation. Twenty-four hours later, the supernatant of the HBV transfected HepG2 (HBV-HepG2) was collected to evaluate the transfection efficiency, and the transfected cells with supernatant HBV DNA > 5.0 × 10^6^ copies/mL were screened with G418 and sub-cultured at a 1:4 ratio for further study.

### Co-culture of NK and HepG2/HBV-HepG2 Cells

Newly revived NK-92 cells (American Type Culture Collection, Manassas, VA, USA) were cultured in α-MEM complete medium (Gibco, Grand Island, NY, USA) containing 12.5% FBS, 12.5% horse serum (Gibco, Grand Island, NY, USA), IL-2 (200 IU/mL, Peprotech, Rocky Hill, NJ, USA), IL-15 (0.5 ng/mL, Peprotech, Rocky Hill, NJ, USA). After the cytotoxicity of NK-92 cells was confirmed by MTT, the cells were divided into two co-cultured groups (Group A and B), which were sub-divided into different subgroups and were stimulated with different interventions: Group A (a. NK + HepG2; b. NK + HepG2 + NKG2DmAb; c. NK + HepG2 + IFN-α; and d. NK + HepG2 + IFN-α + NKG2DmAb); Group B (a. NK + HBV-HepG2; b. NK + HBV-HepG2 + NKG2DmAb; c. NK + HBV-HepG2 + IFN-α; and d. NK + HBV-HepG2 + IFN-α + NKG2DmAb). (IFN-α, Peprotech, Rocky Hill, NJ, USA; NKG2DmAb, R&D, Minneapolis, MN, USA). The final concentrations of IFN-α and NKG2DmAb in the co-culture experiments were 10 ng/mL and 1 μg/mL, respectively. Both were added to the NK cells cultures system 4 h before the co-culture for pre-activating NK-92 cells. The supernatants were collected after 24 h for detection of TNF-α, IFN-γ, perforin and granzyme B levels, and cells were harvested to assess the mRNA and protein expressions of NKG2D and IFN-γ.

### Cytotoxicity Assay

Cytotoxicity of NK cells against HepG2/HBV-HepG2 target cells (T) was analyzed using a CytoTox-Fluor™ Cytotoxicity Assay kit (Promega, Madison, WI, USA), according to the manufacturer’s instructions. Target cells in the logarithmic phase were plated and incubated in 96-well plate for 72 h. NK-92 effector cells (E) were then added to the wells containing the target cells at various T/E ratios (1/2–1/16) for 4 h at 37 °C after adding 100 μL of the Cytotoxicity Assay Reagent per well. In blocking experiments, NK-92 cells were cultured in medium with 1.0 μg/mL of purified anti-human NKG2D antibody (BioLegend, San Diego, CA, USA) for 24 h before incubating with target cells. Control wells contained either only NK cells, only target cells or anti-NKG2D in the well with NK cells. The resulting fluorescence was measured using a fluorometer (485 nmEx/520 nmEm). The percentage of target cells killed by NK-92 cells was calculated as follows: 100 × (sample protease activity − spontaneously released protease activity)/(total maximum protease activity − spontaneously released protease activity).

### RNA Extraction and Real-time PCR Analysis

Total RNA was extracted from the RNAlater-stored liver tissue or cultured cells using Trizol Reagent (Invitrogen, Carlsbad, CA, USA). cDNA synthesis by reverse transcription was performed using PrimeScriptTM RT Kit (Thermo Fermentas, Burlington, ON, Canada) according to the manufacturer’s instructions. Primers used in this study were as follow: NKG2D-Forward: 5′-CGC TGT AGC CAT GGG AAT C-3′; -Reverse: 5′-AAT GTG TAC TAG TCC CAT CCA ATG A-3′; IFN-γ-Forward: 5′-CTC ATG TAA GCC CCC AGA AA-3′; -Reverse: 5′-GCC CAG TTC CTG CAG AGT A G-3′; GAPDH-Forward: 5′-ACC ACA GTC CAT GCC ATC ACT-3′;-Reverse: 5′-TCC ACC ACC CTG TTG CTG TA-3′. Real time quantitative RT-PCR (ABI PRISM 7500; Roche Molecular Systems, Inc., Alameda, CA, USA) was performed using a GoTaq® RT-qPCR System (Promega, Madison, WI, USA) following the manufacturer’s instructions. Real time-PCR reaction for NKG2D and IFN-γ was performed as follows: 95 °C for 5 min followed by 35 cycles of 95 °C for 10 s, 56 °C for 30 s and 68 °C for 45 s. Samples without template and reverse transcriptase were included as negative controls. Relative mRNA levels of NKG2D and IFN-γ were calculated by comparative threshold cycle (Ct) analysis after normalization for the relative amount of GAPDH in the same samples, and were represented as 2^−ΔΔCt^ which were transformed from the initial Ct value.

### Western Blotting Analysis

Total proteins were extracted from cultured cells in each group with RIPA lysis buffer (Sigma, St. Louis, MO, USA), and concentrations were determined using a BCA protein assay kit (Thermo Scientific, Rockford, IL, USA) according to the manual. 20 μg of protein were separated by 12% SDS-PAGE and then was transferred from gel to PVDF transfer membrane (Thermo Scientific, Rockford, IL, USA) by semi-dry transfer method. Following protein transfer, PVDF membranes were rinsed with TBS, placed in TBS/T blocking buffer containing 5% (w/v) skimmed milk powder and incubated with rabbit-anti-human NKG2D (1:1000 dilution, Abcam, Cambridge, MA, USA), IFN-γ (1:200 dilution, Boster Biotechnology, Wuhan, China) and β-actin independently overnight at 4 °C. Then membrane was rinsed with TBST and further incubated with HRP-conjugated anti-rabbit secondary antibodies (1:10000 dilution, ZSGB Biotech, Beijing, China) at room temperature for one hour. Image Pro Plus software (Media Cybernetics, Silver Spring, MD, USA) was used to analyze the ratio of target protein blotting relative to β-actin.

### Enzyme-linked Immunosorbent Assay

Concentrations of IFN-γ (No. 201-TA, sensitivity 8 pg/mL), TNF-α (No. DTA00C, sensitivity 5.5 pg/mL), perforin (No. ab46068, sensitivity <20 pg/mL) and granzyme B (No. ab46142, sensitivity <20 pg/mL) in serum and supernatants were measured with sandwich ELISA using a commercial ELISA kit (IFN-γ and TNF-α from R&D, Minneapolis, MN, USA; perforin and granzyme B from Abcam, Cambridge, MA, USA) per manufacturer’s recommended protocol. A standard curve was calculated by plotting optical density *versus* the log concentration.

### Statistical Analysis

Statistical analysis was performed with IBM SPSS Statistics version 17.0 from SPSS Inc. (Chicago, IL, USA). Normally distributed continuous variables were analyzed using one-way ANOVA, followed by Student-Newman-Keuls *q* test for evaluating variances between each two groups. For non-normally distributed or variance homogenous data, statistical differences were analyzed using nonparametric Kruskal-Wallis *H* test, followed by Nemenyi test for pairwise comparisons between two groups. Pearson Chi-square test or Fisher’s exact test was used to analyze categorical variables as appropriate. A two-sided *P* value of <0.05 was considered statistically significant.

## Electronic supplementary material

Supplementary Information

## References

[1] Chisari FV, Isogawa M, Wieland SF (2010). Pathogenesis of hepatitis B virus infection. Pathol Biol (Paris).

[2] Xu D (2006). Circulating and liver resident CD4+ CD25+ regulatory T cells actively influence the antiviral immune response and disease progression in patients with hepatitis B. J Immunol.

[3] Dunn C (2007). Cytokines induced during chronic hepatitis B virus infection promote a pathway for NK cell-mediated liver damage. J Exp Med.

[4] Szabo G, Mandrekar P, Dolganiuc A (2007). Innate immune response and hepatic inflammation. Semin Liver Dis.

[5] Zhang Z (2007). Increased infiltration of intrahepatic DC subsets closely correlate with viral control and liver injury in immune active pediatric patients with chronic hepatitis B. Clin Immunol.

[6] Zhang Z (2008). Severe dendritic cell perturbation is actively involved in the pathogenesis of acute-on-chronic hepatitis B liver failure. J Hepatol.

[7] Marras F, Bozzano F, De Maria A (2011). Involvement of activating NK cell receptors and their modulation in pathogen immunity. J Biomed Biotechnol..

[8] Zhang Z (2011). Hypercytolytic activity of hepatic natural killer cells correlates with liver injury in chronic hepatitis B patients. Hepatology.

[9] Peppa D (2013). Up-regulation of a death receptor renders antiviral T cells susceptible to NK cell-mediated deletion. J Exp Med.

[10] Koumbi L (2016). Hepatitis B viral replication influences the expression of natural killer cell ligands. Ann Gastroenterol.

[11] Bauer S (1999). Activation of NK cells and T cells by NKG2D, a receptor for stress-inducible MICA. Science.

[12] Jamieson AM (2002). The role of the NKG2D immunoreceptor in immune cell activation and natural killing. Immunity.

[13] Chavez-Blanco, A. *et al*. Viral inhibitors of NKG2D ligands for tumor surveillance. Expert Opin Ther Targets 1–13 (2016). 10.1080/14728222.2016.1202928 27322108

[14] Pollicino T, Koumbi L (2015). Role natural killer group 2D-ligand interactions in hepatitis B infection. World J Hepatol.

[15] Chen Y, Sun R, Jiang W, Wei H, Tian Z (2007). Liver-specific HBsAg transgenic mice are over-sensitive to Poly(I:C)-induced liver injury in NK cell- and IFN-gamma-dependent manner. J Hepatol.

[16] Chen Y (2007). Increased susceptibility to liver injury in hepatitis B virus transgenic mice involves NKG2D-ligand interaction and natural killer cells. Hepatology.

[17] Zou Y (2010). Increased killing of liver NK cells by Fas/Fas ligand and NKG2D/NKG2D ligand contributes to hepatocyte necrosis in virus-induced liver failure. J Immunol.

[18] Dong S, Geng L, Shen MD, Zheng SS (2015). Natural Killer Cell Activating Receptor NKG2D Is Involved in the Immunosuppressive Effects of Mycophenolate Mofetil and Hepatitis B Virus Infection. Am J Med Sci.

[19] Liu YX (2008). Effect of hepatitis B virus C protein on function of natural killer cell in NK-92 cells. Zhonghua Shi Yan He Lin Chuang Bing Du Xue Za Zhi.

[20] Ahmad A, Alvarez F (2004). Role of NK and NKT cells in the immunopathogenesis of HCV-induced hepatitis. J Leukoc Biol.

[21] Yang Y, Han Q, Zhang C, Xiao M, Zhang J (2016). Hepatitis B virus antigens impair NK cell function. Int Immunopharmacol.

[22] Stegmann KA (2012). Interferon alpha-stimulated natural killer cells from patients with acute hepatitis C virus (HCV) infection recognize HCV-infected and uninfected hepatoma cells via DNAX accessory molecule-1. J Infect Dis.

[23] Zheng Q (2015). Activated natural killer cells accelerate liver damage in patients with chronic hepatitis B virus infection. Clin Exp Immunol.

[24] Heiberg IL (2015). Defective natural killer cell anti-viral capacity in paediatric HBV infection. Clin Exp Immunol.

[25] Beziat V (2012). CMV drives clonal expansion of NKG2C+ NK cells expressing self-specific KIRs in chronic hepatitis patients. Eur J Immunol.

[26] Li F (2013). Blocking the natural killer cell inhibitory receptor NKG2A increases activity of human natural killer cells and clears hepatitis B virus infection in mice. Gastroenterology.

[27] Baron JL (2002). Activation of a nonclassical NKT cell subset in a transgenic mouse model of hepatitis B virus infection. Immunity.

[28] Liu, F. *et al*. Lower number and decreased function of natural killer cells in hepatitis B virus related acute-on-chronic liver failure. Clin Res Hepatol Gastroenterol (2016). 10.1016/j.clinre.2016.01.004 27053076

[29] Huang M, Sun R, Wei H, Tian Z (2013). Simultaneous knockdown of multiple ligands of innate receptor NKG2D prevents natural killer cell-mediated fulminant hepatitis in mice. Hepatology.

[30] Vilarinho S, Ogasawara K, Nishimura S, Lanier LL, Baron JL (2007). Blockade of NKG2D on NKT cells prevents hepatitis and the acute immune response to hepatitis B virus. Proc Natl Acad Sci USA.

[31] Takahashi E (2007). Induction of CD16+ CD56bright NK cells with antitumour cytotoxicity not only from CD16- CD56bright NK Cells but also from CD16- CD56dim NK cells. Scand J Immunol.

[32] Liaw YF (2008). Asian-Pacific consensus statement on the management of chronic hepatitis B: a 2008 update. Hepatol Int..

[33] Sarin SK (2009). Acute-on-chronic liver failure: consensus recommendations of the Asian Pacific Association for the study of the liver (APASL). Hepatol Int.

[34] Wang Y (2014). Predictive value of interferon-gamma inducible protein 10 kD for hepatitis B e antigen clearance and hepatitis B surface antigen decline during pegylated interferon alpha therapy in chronic hepatitis B patients. Antiviral Res.

[35] Li D (2011). Core-APOBEC3C chimerical protein inhibits hepatitis B virus replication. J Biochem.

[36] Sun D, Nassal M (2006). Stable HepG2- and Huh7-based human hepatoma cell lines for efficient regulated expression of infectious hepatitis B virus. J Hepatol.

[37] Huang JH (2007). Identification of the HIV-1 gp41 core-binding motif in the scaffolding domain of caveolin-1. J Biol Chem.

