# Supplementary material for: NKG2D modulates aggravation of liver inflammation by activating NK cells in HBV infection
Source: Sci Rep. 2017 Mar 7;7:88. doi: 10.1038/s41598-017-00221-9 (PMC5427972; doi:10.1038/s41598-017-00221-9)
Supplement: Supplementary file 1 — Supplementary Information [file 41598_2017_221_MOESM1_ESM.pdf]

NKG2D modulates aggravation of liver inflammation by activating NK cells in HBV infection

Yadong Wang[1], Wei Wang[1], Chuan Shen[1], Yong Wang[2], Mingjing Jiao[1], Weiyan Yu[1], Hongzhu Yin[1], Xiaobo Shang[1], Qianfei Liang[1], Caiyan Zhao[1*]

Suppl. Figure legends

Suppl. Figure 1. The percentage of peripheral NK cells ($CD3^{-}$ $CD56^{+}$) in PBMC of ACLF patients, CHB patients, AsC patients, and healthy controls. The $CD3^{-}CD56^{+}$ NK cells are gated from lymphocyte subsets of isolated PBMCs. (A) Representative dot plots are obtained by flow cytometry using antibodies against both CD3 and CD56; (B) Frequency of $CD3^{-}CD56^{+}$ NK cells from subjects in four studied groups. Compare with HC group, ◆◆◆P<0.01; Compare with AsC group, ★★P<0.05.

Suppl. Figure 2. HE staining of hepatic tissues in each group (200×). (a): Healthy control group; (b): Immuno-torlerance group (AsC); (c): Immuno-active group (CHB); (d): HBV-related ACLF group; Compare with HC group, ◆◆◆*P*<0.01; Compare with AsC group, ★★★*P*<0.01; Compare with CHB group, ▲▲▲*P*<0.01.

Suppl. Figure 3. Immunohistochemistry double stain of intrahepatic NK cells. Representative graphs of intrahepatic NK ($CD3^{-}$ $CD57^{+}$) cells expression in different group patients ((a-d) 200×, (e) 400×). NK cells were mainly localized in the inflammatory areas or occasionally scattered in hepatic sinusoids. (a): Healthy control group; (b): Immuno-torlerance group (AsC); (c): Immuno-active group (CHB); (d and e): HBV-related ACLF group; Compare with HC group, ◆◆◆*P*<0.01; Compare with AsC group, ★★★*P*<0.01;

Compare with CHB group, ▲▲▲$P<0.01$. →NK cells ($CD3^{-}CD57^{+}$); →NKT cells ($CD3^{+}$ $CD57^{+}$).

Suppl. Figure 4. The NK cells and HepG2 coculture at 24h. (a) NK cells (× 200); (b) NK cells (× 400); (c) HepG2 cells (× 200); (d) NK cells + HepG2 cells (× 100); (e) NK cells + HepG2 cells (× 200); (f) NK cells + HBV-HepG2 cells (× 400)

Suppl. Figures

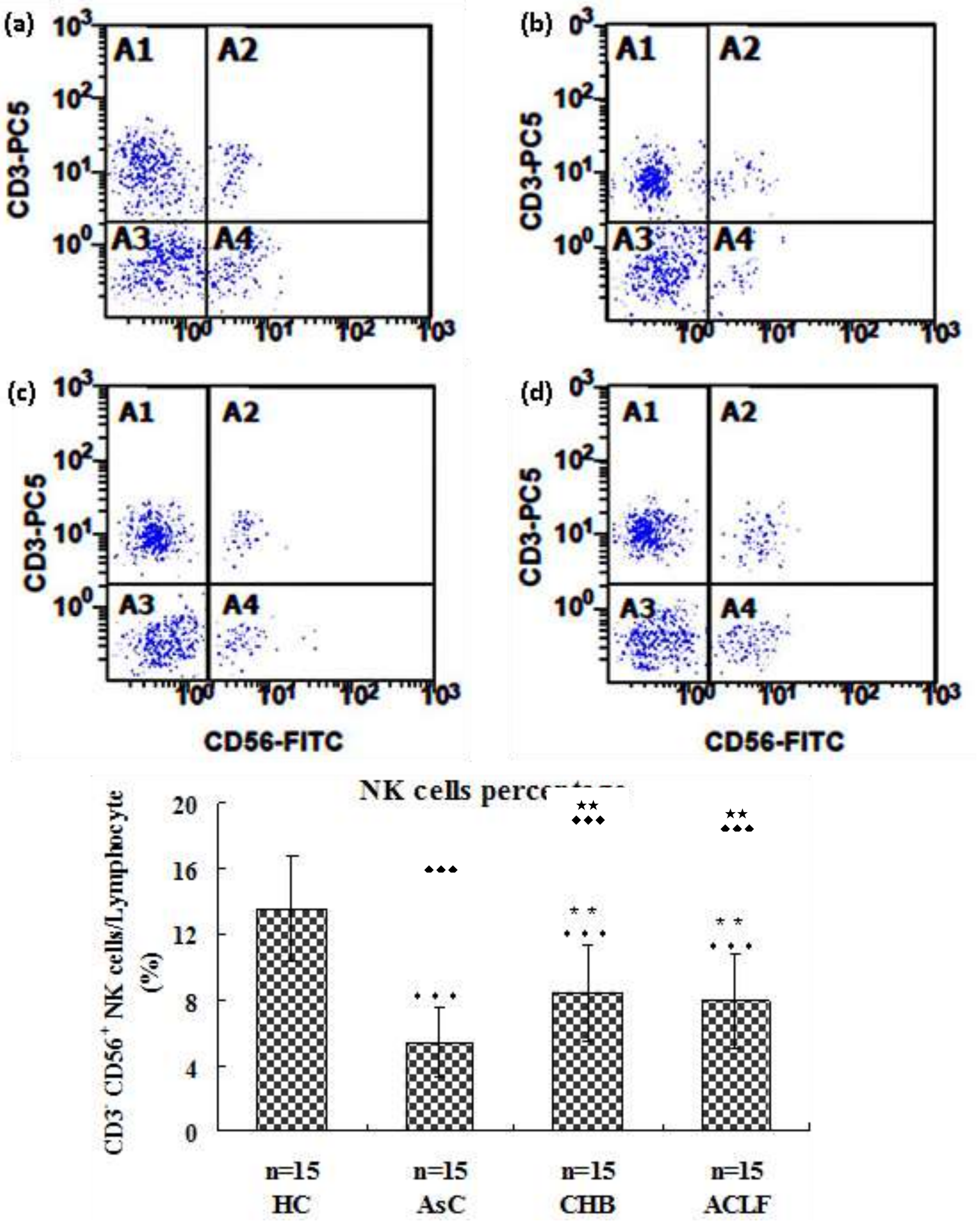

Suppl. Figure 1 The percentage of NK cells (CD3⁻ CD56⁺) in PBMC

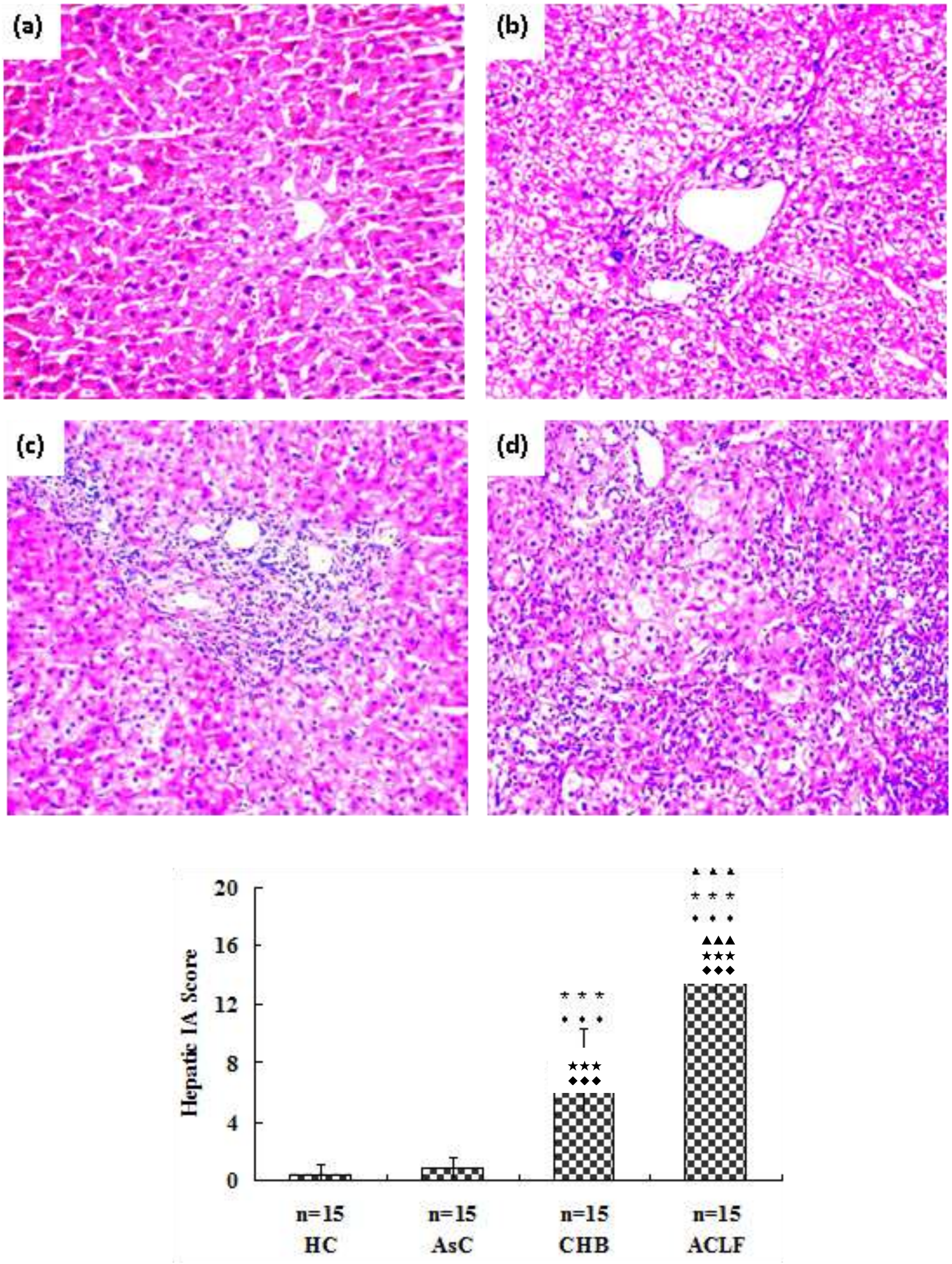

Suppl. Figure 2 HE staining of hepatic tissues

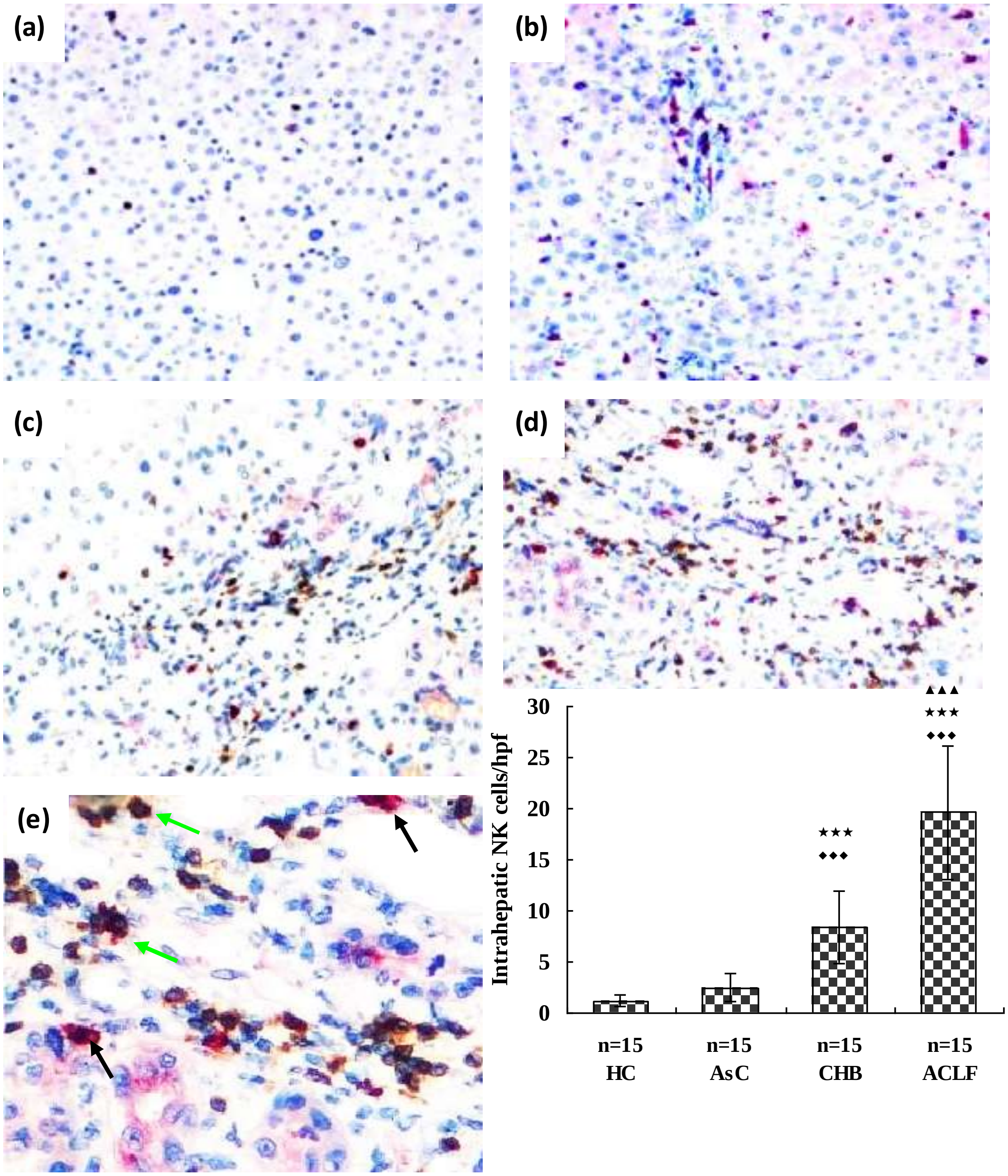

Suppl. Figure 3 Immunohistochemistry double stain of intrahepatic NK cells

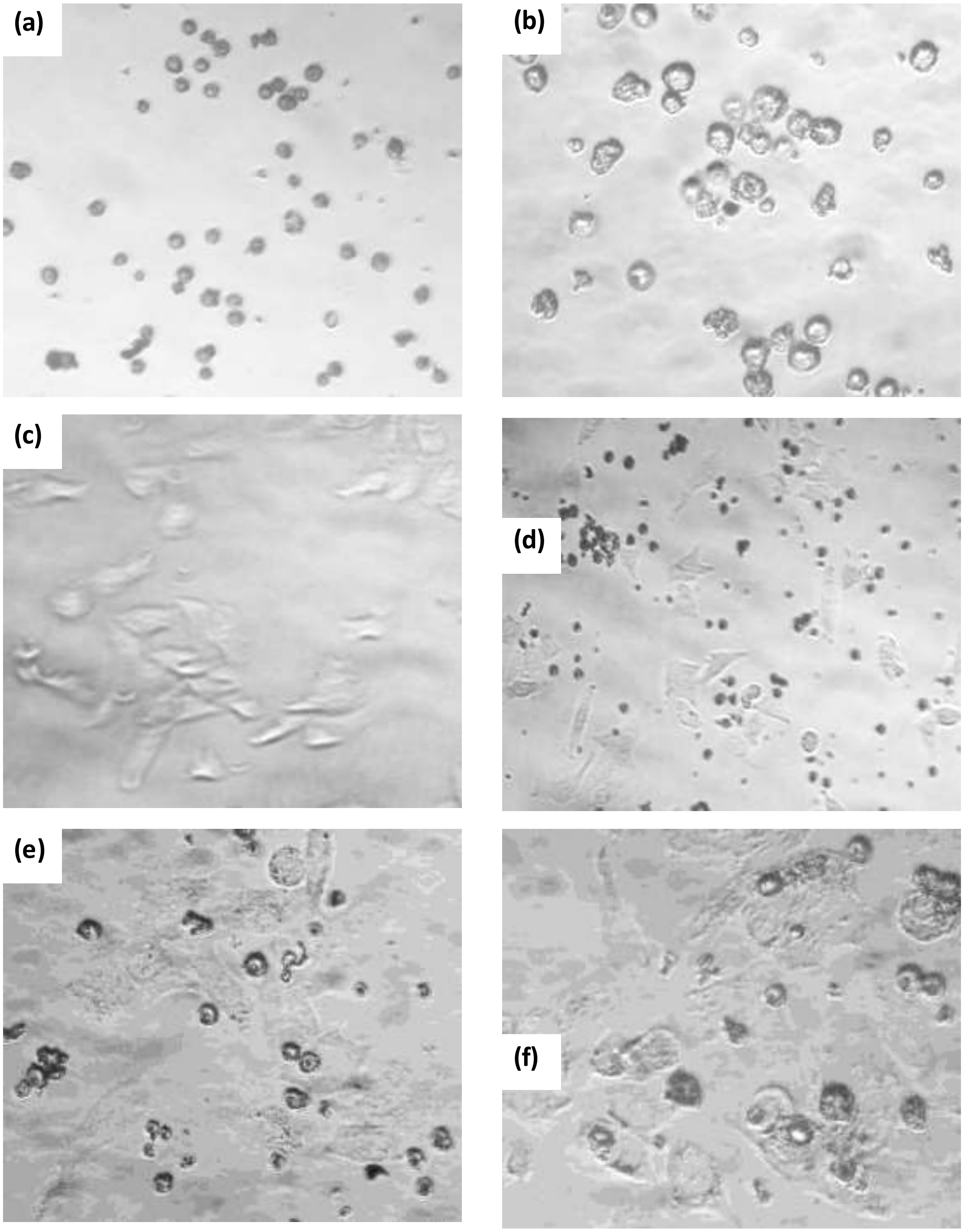

Suppl. Figure 4 The NK cells and HepG2 coculture at 24h
